# Characterization of Bone Marrow and Wharton’s Jelly Mesenchymal Stromal Cells Response on Multilayer Braided Silk and Silk/PLCL Scaffolds for Ligament Tissue Engineering

**DOI:** 10.3390/polym12092163

**Published:** 2020-09-22

**Authors:** Xing Liu, Adrien Baldit, Emilie de Brosses, Frédéric Velard, Ghislaine Cauchois, Yun Chen, Xiong Wang, Natalia de Isla, Cédric Laurent

**Affiliations:** 1 CNRS UMR 7365 IMoPA - Université de Lorraine, 54500 Vandœuvre-lès-Nancy, France; xing.liu@univ-lorraine.fr (X.L.); ghislaine.cauchois@univ-lorraine.fr (G.C.); xiong.wang@univ-lorraine.fr (X.W.); Natalia.De-Isla@univ-lorraine.fr (N.d.I.); 2 Université de Lorraine, CNRS, Arts et Métiers ParisTech, LEM3, F-57000 Metz, France; adrien.baldit@univ-lorraine.fr (A.B.); emilie.de-brosses@univ-lorraine.fr (E.d.B.); 3 Université Reims Champagne-Ardenne, BIOS EA 4691 1, avenue du Maréchal Juin, 51095 Reims, France; frederic.velard@univ-reims.fr; 4 Department of Biomedical Engineering, School of Basic Medical Science, Wuhan University, Wuhan 430000, China; yunchen@whu.edu.cn; 5 Université de Lorraine, CNRS, Arts et Métiers ParisTech, LEM3, F-54000 Nancy, France

**Keywords:** stromal cells, ligament tissue engineering, braided scaffold, silk, PLCL, biocompatibility

## Abstract

(1) Background: A suitable scaffold with adapted mechanical and biological properties for ligament tissue engineering is still missing. (2) Methods: Different scaffold configurations were characterized in terms of morphology and a mechanical response, and their interactions with two types of stem cells (Wharton’s jelly mesenchymal stromal cells (WJ-MSCs) and bone marrow mesenchymal stromal cells (BM-MSCs)) were assessed. The scaffold configurations consisted of multilayer braids with various number of silk layers (*n* = 1, 2, 3), and a novel composite scaffold made of a layer of copoly(lactic acid-*co*-(e-caprolactone)) (PLCL) embedded between two layers of silk. (3) Results: The insertion of a PLCL layer resulted in a higher porosity and better mechanical behavior compared with pure silk scaffold. The metabolic activities of both WJ-MSCs and BM-MSCs increased from day 1 to day 7 except for the three-layer silk scaffold (S3), probably due to its lower porosity. Collagen I (Col I), collagen III (Col III) and tenascin-c (TNC) were expressed by both MSCs on all scaffolds, and expression of Col I was higher than Col III and TNC. (4) Conclusions: the silk/PLCL composite scaffolds constituted the most suitable tested configuration to support MSCs migration, proliferation and tissue synthesis towards ligament tissue engineering.

## 1. Introduction

Ligaments consist of fascicles of dense connective tissue, and play a key role in supporting internal organs and connecting bones together within joints [1,2]. Decades of research have resulted in significant knowledge regarding the biological and mechanical properties of a ligament and the associated grafts used in ligamentoplasty, and also have emphasized promising perspectives coming from the field of tissue engineering [3,4,5,6]. However, the high incidence of ligament disorders together with its inherent poor self-regeneration capacity still constitutes persistent challenges. One of the milestone for the design of a tissue-engineered ligament is to propose and manufacture a biomaterial-based construct that could mimic the biological properties, mechanical strength and microstructure of the native ligament tissue [3]. Indeed, it has been often pointed out that one of the milestone in tissue engineering lies in fostering biomaterials that can restore both biological and mechanical functions [7]. Despite the number of studies that have already been reported, there is still an urgent demand to propose and optimize cell-scaffold constructs with satisfying mechanical properties, biodegradability, morphological and biological properties for ligament tissue regeneration.

Among the reported candidate biomaterials for ligament tissue engineering, silk has gained a growing interest. It is a natural protein-based polymer, and it has been widely used as a constitutive material in tissue engineering due to its biocompatibility, mechanical properties and low immunogenicity [8]. Numerous studies involve silk or silk-based composites (associated with collagen, polyurethane, poly(lactic-co-glycolic acid), RADA16, gelatin, etc.) for ligament tissue engineering [8]. Altman et al [9] firstly demonstrated that a silk fiber-based scaffold could promote bone marrow stromal cells attachment, proliferation and differentiation towards ligament lineage, and particularly emphasized the interest of mechanical stimulation for the regeneration of ligament-like tissue. More recently, Ouyang et al. [10,11,12,13] reported promising tissue engineering studies applied to musculoskeletal tissues such as rotator cuff [13], Achilles tendon [11] medial collateral ligament (MCL) [12] and anterior cruciate ligament (ACL) [10] based on a knitted silk–collagen sponge. These silk/collagen sponges have been shown to enhance ligament repair by inducing more collagen matrix synthesis, increasing mechanical properties, resulting in the regeneration of a microstructure close to the native tissue. After 18 months of in vivo implantation [10], silk has shown no obvious sign of degradation. This slow degradation rate has led the FDA [14] to classify silk as a non-degradable biomaterial.

In our previous study [15], PLCL (poly-(l-lactide-*co*-ε-caprolactone)) has been used to construct a multilayer braided scaffold for tissue engineering application. The PLCL scaffold showed satisfying initial mechanical properties and biocompatibility, and encouraged the adhesion, migration, proliferation of cells as well as tissue regeneration [15,16]. Moreover, this scaffold exhibited high flexibility and tunable elasticity, showing potential application towards ligament regeneration. Nevertheless, our team has recently reported [16] that PLCL may present a brittle mechanical response after 48 days of in vitro degradation, resulting in a risk or premature failure of the scaffold if the tissue regeneration is not sufficient in the early weeks of implantation. Based on previous studies that have confirmed the suitability of both PLCL and silk towards ligament tissues, their association into composite scaffolds may constitute a means to achieve the suited mechanical behavior and degradation properties in the challenge of proposing a solution for ligament tissue engineering. As far as a cell source is concerned, since there is not a clear consensus on the most suited type of cells for ligament tissue engineering [17], two types of mesenchymal stromal cells (MSCs) were tested and compared in our previous study [15] (namely Wharton’s jelly mesenchymal stromal cells (WJ-MSCs) and bone marrow mesenchymal stromal cells (BM-MSCs)) with no significant differences. Both were selected as privileged cell sources to interact with PLCL-based scaffold due to their self-renewability, differentiation potential and bioactive molecule secretion activity, and the question of which of these cell types is the more adapted to ligament tissue engineering is still open. Recent research [18] have indeed emphasized the regenerative potential of cells based on an umbilical cord, while bone marrow-based cells are used in most studies.

In the present study, different multilayer braided scaffold configurations made of silk alone (with *n* = 1, 2, 3 layers) or silk combined with PLCL into a silk/PLCL composite scaffold were characterized and compared for two different cell types (BM-MSCs and WJ-MSCs). Studies included the characterization of scaffold mechanical and morphological properties, as well as the evaluation of MSCs proliferation, morphology and location, the distribution of live cells, cell migration and extracellular matrix synthesis. The following sections describe the methods associated with these different characterizations and the obtained results and conclusions are then provided concerning the relevance of the different proposed scaffolds for ligament regeneration.

## 2. Materials and Methods

### 2.1. Preparation of Silk and the Silk/PLCL Multilayer Braided Scaffold

Commercial raw silk fibers (Bombyx mori silk on the form of 20/22D yarns) were selected (Trudel Limited, Zurich, Switzerland). Silk yarns were firstly wound around bobbins, and the sericin was removed by degumming the silk in 0.02 M Na_2_CO_3_ solution at 100 ℃ for more than 1.5 h and them rinsing overnight with water. At the end of this process, silk was washed three times with distilled water. Sixteen bobbins were installed on a braiding machine (Composite and Wire machinery, United States) as previously reported (Laurent et al., 2011) to fabricate multilayer braided scaffolds. Briefly, the process consisted of building 16-wires braid using the maypole braiding machine, and multilayer scaffolds were simply processed by repeating the braiding motion around existing braids. Firstly, silk braided scaffolds with 1, 2 and 3 layers (hereafter called S1, S2 and S3) were thus built following this process. Secondly, by alternating the bobbins used between two layers, a novel silk/PLCL composite scaffold was processed by placing a layer of braided PLCL wires between two layers of braided silk wires. This silk–polymer scaffold has been hereafter called SP. For all configurations, scaffold extremities were sutured with silk fibers, and then the assemblies were cut into 1 cm-long scaffolds for biological studies and 4 cm-long scaffolds for mechanical and morphological studies. The chemical analysis of PLCL fibers by Fourier transform infrared (FTIR) spectroscopy have been detailed in our previous study [15], while numerous chemical analyses of raw silk from *Bombyx mori* are available in the literature (e.g., [19]).

### 2.2. Characterization of Physical Properties of Silk and Silk/PLCL Scaffolds

#### 2.2.1. Morphological Characterization by Scanning Electronic Microscopy (SEM)

In order to observe the global morphology of the proposed scaffold configurations, SEM observations were firstly performed. The scaffold was subjected to a series of gradient alcohol solutions baths (two repetitions of 10 min at 50°, 10 min at 70°, 10 min at 90° and 10 min at 100°) to achieve dehydration. Secondly, a gold coating was applied before SEM observations (JEOL 5400 LV).

#### 2.2.2. Morphological Characterization and Porosity Calculation by µCT

**Figure 1:**
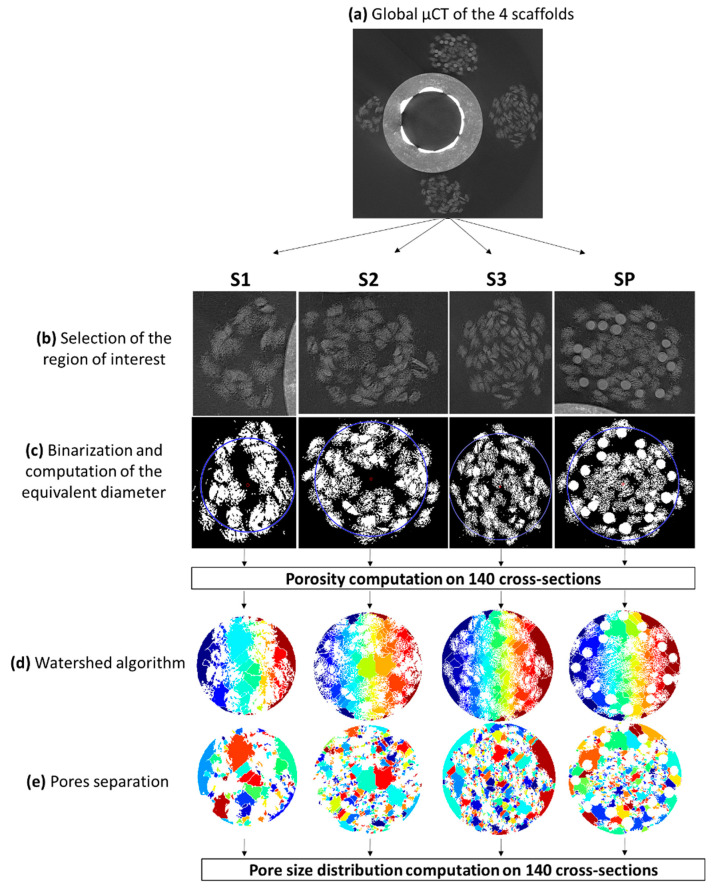
Image processing routine for post-treatment of µCT cross-sections for the four scaffold configurations (S1: one-layer silk scaffold; S2: two-layer silk scaffold; S3: three-layer silk scaffold; SP: silk/PLCL composite scaffold).

The four scaffolds (S1, S2, S3 and SP) were scanned at 50 kV and 50 µA using a nanotomograph (EasyTom Nano, RX Solutions, Chavanod France). A custom-made device was designed to achieve a good resolution (4.89 µm/voxels) while scanning the four configurations in a single acquisition, in order to avoid the differences of the X-ray flux between two acquisitions, and then generate differences on the segmentations. In the middle part of the scaffolds 140 slices were selected and post-processed using custom Matlab routines explained hereafter. After binarization of the image, the center of gravity of the images was computed. The diameter of the circle (centered at the center of gravity) containing 99% of the non-zero values of the images was defined as the equivalent scaffold diameter, which was then computed for each cross-section (Figure 1). Standard watershed algorithms were then applied to define pores within this circle, in order to finally compute global pore size distribution.

#### 2.2.3. Mechanical Characterization

The tensile response of S1, S2, S3 and SP scaffolds were assessed and compared using routine tensile tests performed in a physiological environment by immersion into water at 37 °C. Scaffolds were subject to increasing loading–unloading cycles using a tensile machine (Zwick Roell Z0.50 TN with a load cell XforceP of 100 N) and custom grips to screw the scaffolds. After measuring the distance between grips to define the initial scaffold length, displacements were applied at a speed of 0.1 mm/s in such a way that it corresponded to 1%, 3%, 5% and 10% of the initial scaffold length. Scaffold properties were determined from the post-treatment of recorded forces and displacements during the tensile test, involving the calculation of strain and stress. For each loading cycle and each scaffold configuration, the two following moduli were computed: the “toe” modulus was defined as the slope at the origin of the stress–strain response, while the “linear” modulus was defined was the slope of the stress–strain response when the prescribed strain was reached. 

### 2.3. Evaluation of Biocompatibility of MSCs on Scaffolds 

#### 2.3.1. MSC Isolation and Expansion

WJ-MSCs and BM-MSCs were isolated and expanded according to a previously described method [15]. α-MEM (Lonza) medium, containing 10% fetal calf serum (FCS, Dominique Dutscher), 2 mM L-glutamine (Sigma), 100 U/mL penicillin /streptomycin (Gibco) and 1 μg/mL amphoterin B (Gibco) was used for cell culture. MSCs were incubated at 37 °C, 5% CO_2_, 90% humidity and the medium was changed twice a week.

#### 2.3.2. MSCs Culture on Scaffolds

MSCs were detached at passage 2 (P2) with a 70–80% confluence. Scaffolds were sterilized with UV radiation for 30 min each side. As described in [5], cells were seeded onto the scaffolds by depositing drops of cell suspension within the periphery of the scaffolds. Due to the pore size gradient offered by the scaffold architecture, the cell suspension immediately penetrated the scaffold center and spread along the scaffold. The volumes of cell suspension were optimized for each scaffold configuration and finally 3 × 10^5^ cells were suspended into 20 µL for S1, 40 µL for S2, 60 µL for S3 and 30 µL for SP in such a way that the drops spread along the scaffold. MSC scaffolds were maintained 1 h in the incubator for initial cellular adhesion before supplying 1 mL of the α-MEM complete medium containing 100 μg/mL of ascorbic acid (Sigma), and then cultured for 2 weeks. Medium was changed twice a week.

#### 2.3.3. Metabolic Activity of MSCs on Scaffolds

MSC metabolic activity was evaluated by Alamar Blue (AB) tests (Thermo Fisher Scientific, Waltham, Massachusetts, United States MSC scaffolds were firstly washed with PBS for 2 times, then 1 mL of 10% AB working solution was added to each scaffold and incubated for 4 h. The absorbance was measured at 570 nm and 600 nm (*n* = 3) on day 1, 3, 5 and 7. AB working solution was considered as the negative control. The percentage of Alamar Blue reagent reduction was determined following the manufacturer’s protocol. 

#### 2.3.4. Distribution and Viability of MSCs on Scaffolds

MSCs distribution on scaffolds by SEM: After 2 weeks of culture, MSC scaffolds were firstly washed with PBS for 2 times and fixed with 2.5% glutaraldehyde (*v*/*v* in PBS) at 4 °C overnight. After 2 distilled water rinses, MSC scaffolds were dehydrated in graded alcohol solution baths (50%, 70%, 90% and 100% 2 times) for 10 min. MSC scaffolds were finally desiccated in a drop of hexamethyldisilazane (HMDS, Sigma-Aldrich, St. Louis, Missouri, United States). After air-drying at room temperature, samples were sputtered with a thin gold-palladium film using a JEOL ion sputter JFC 1100 instrument. MSC scaffolds were observed using a Schottky field emission scanning electron microscope (JEOL JSM-7900F from PICT-URCA platform, Tokyo, Japan). Images were obtained at a primary beam energy of 2 kV (SM-EXG65 electron emitter, JEOL, Tokyo, Japan).

Staining of live MSCs on scaffolds: Viable MSCs on scaffolds were visualized due to calcein AM (BioLegend) staining. The working solution was prepared as followed: 4 µL of 1 mM calcein AM was added to 1 mL of α-MEM medium without phenol red. After homogeneous mixing of the solution, MSC scaffolds were firstly rinsed twice with α-MEM medium without phenol red. The scaffold was incubated at 37 °C for 45 min after the addition of 250 µL of staining working solution. MSC scaffolds were finally observed under fluorescence microscopy (ex/em Calcein: 494/517 nm). 

#### 2.3.5. MSCs Chemotaxis Induced by Scaffolds

Boyden chamber assays were used to evaluate the simulation of MSC migration induced by the different scaffold configurations. This was realized by the use of a transwell (Corning^®^ FluoroBlok™, Sigma-Aldrich, St. Louis, MO, United States) with an 8 μm pore fluorescence blocking PET track-etched membrane insert. For each scaffold, 10 mg of constitutive material was processed into powder and sterilized by the use of a 30 min period under UV radiation. For SP scaffold, the silk/PLCL ratio (in mass) was equivalent to the proportion of silk and PLCL fibers within the SP scaffold. A coating of 100 µL collagen was applied for 30 min on the downside of the membrane.

Cells were firstly starved for a period of 24 h, and then 1.5 × 10^5^ MSCs were added to 200 µL of the α-MEM medium without FCS, which was added into each reservoir. α-MEM medium with FCS was added to the four different powders in a proportion of 500 µL/well in 24-well chamber, and maintained during 24 h. Calcein AM (Thermo Fisher Scientific; 1/1000) staining was added to the cells attached on the membrane in order to perform fluorescence microscopy (Leica SP5 microscope TCS SP5 II, Leica, Solms, Germany).

#### 2.3.6. Histology and Immunohistochemistry

MSC scaffolds (*n* = 3) were harvested and fixed in 1% paraformaldehyde fix solution after 14 days of culture. After dehydrating of the samples and embedding them in paraffin, 5 µm pieces were obtained using microtome and stained with both Red Sirius (RS) and hematoxylin/eosin/safran (HES) for histological evaluation. The Mouse and Rabbit Specific HRP/DAB (ABC) Detection IHC Kit (Abcam, ab64264) were used to evaluate the expression of Col I, Col III and TNC by immunohistochemistry. To do so, we firstly deparaffinized and rehydrated the samples, pepsin was added for antigen retrieval for 30 min and slides were then washed with PBS twice. Endogenous peroxidase was blocked by immersing the samples into hydrogen peroxide for 10 min. Samples were washed twice with PBS and a nonspecific background was blocked before the incubation with the first antibody using protein block solution. Samples were then incubated for 1 h with monoclonal antibodies specific to TNC (ab6393, Abcam), Col I (ab88147, Abcam) and Col III (C7805, Sigma) according to the manufacturer’s protocol, and then washed three times in PBS. The resulting samples were incubated for 10 min at room temperature with biotinylated goat antipolyvalent before being washed twice with PBS. The same operation was performed with streptavidin peroxidase for 10 min. Samples were finally washed four times with PBS, and a solution of DAB diluted at 1/50 was used as a chromogenic agent. Hematoxylin was used as the counterstain.

### 2.4. Statistical Analysis

All the data were considered in terms of mean ± standard deviation (SD) and a one-way ANOVA analyses were performed to assess the statistical analysis by using GraphPad Prism 6. Value of *p* < 0.05 was defined as statistical significance.

## 3. Results

### 3.1. Physical Properties of Silk-Based Braided Scaffolds

#### 3.1.1. Morphological Characterization of Scaffolds by SEM

From SEM observations, the different scaffold configurations presented a braided porous structure, with both the silk fibers and silk yarns clearly distinguishable. A higher external diameter together with a denser structure was observed for increasing the numbers of layers. The measured diameter of silk yarn was about 281 ± 43 µm, while PLCL fibers were extruded with a 170 µm diameter. In the novel silk/PLCL composite scaffold, the PLCL fibers were clearly observed and distinguished from the silk yarns and fibers, and were clearly located in the middle of two silk layers.

#### 3.1.2. Porosity and Pore Size

Using µCT analysis, we evaluated the pore size distribution and porosity of the silk (S1, S2 and S3) and silk/PLCL (SP) scaffolds. In line with morphological observation, compaction of the scaffolds increased with the number of layers (computed porosities were 0.85 ± 0.04, 0.77 ± 0.02 and 0.53 ± 0.02 respectively for S1, S2 and S3). Comparison between both the three layer scaffolds (S3 and SP) indicated that the addition of a PLCL layer induced an increased porosity (0.74 ± 0.02). Pore size distribution was evaluated on µCT images (cross section) using watershed algorithms, and no significant differences were highlighted between the scaffolds. Pore size ranged from 20 to 150 µm and exhibited a typical Weibull distribution, without any significant differences observed among S1, S2, S3 and SP (average values were 29.5 ± 18.2 µm, 28.6 ± 19.2 µm, 30.2 ± 25.5 µm and 28.7 ± 22.1 µm respectively).

#### 3.1.3. Mechanical Characterization

In order to measure force–displacement responses, S1, S2, S3 and SP were subjected to loading–unloading cycles as described previously. For a given prescribed strain of 10%, the values of associated forces for S1, S2, S3 and SP were respectively 4.1 N, 4.2 N, 10.0 N and 6.3 N. When testing the scaffolds up to rupture (data not reported in the present study), the admissible strain was significantly higher for the SP configuration because of the deformability of PLCL at 37 °C, resulting in a very large strain to rupture. Tangent linear moduli at a 10% strain were respectively 65.1, 39.5, 99.6 and 55.3 MPa for S1, S2, S3 and SP respectively, while initial toe moduli were 4.7, 3.0, 3.2 and 3.7 MPa respectively and tended to zero as the prescribed strain increased, indicating a clearly convex non-linear stress–strain curve with a large toe region.

### 3.2. Metabolic Activity of MSCs on Scaffolds

**Figure 2:**
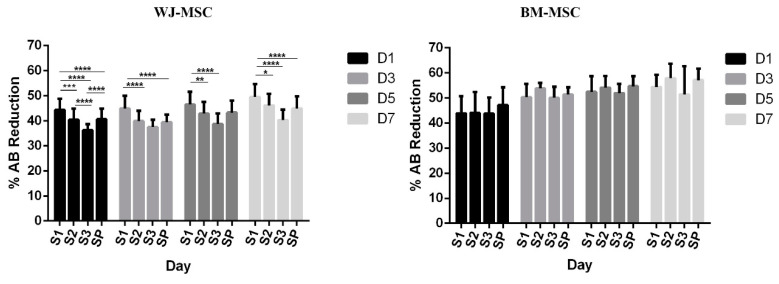
Metabolic activity of Wharton’s jelly mesenchymal stromal cells (WJ-MSCs, left) and bone marrow mesenchymal stromal cells (BM-MSCs, right) on silk and silk/PLCL scaffolds quantified by Alamar Blue assay. (S1: one-layer silk scaffold; S2: two-layer silk scaffold; S3: three-layer silk scaffold; SP: silk/PLCL composite scaffold). Signs “*” indicate the statistical significance.

To assess the biocompatibility of the scaffolds, S1, S2, S3 and SP were seeded with both BM-MSCs and WJ-MSCs, and metabolic activities were then measured by Alamar Blue assay after 1, 3, 5 and 7 days in culture. Figure 2 evidenced an increase from 44.4% ± 4.3%, 40.4% ± 4.4%, 36.3% ± 2.4% and 40.6% ± 4.2% for S1, S2, S3 and SP respectively, to 49.5% ± 5.1%, 46.1% ± 4.6%, 40.3% ± 4.2% and 44.9% ± 4.8% between the first and the seventh day for WJ-MSCs. A similar trend was noticed for BM-MSCs with an increase from 43.9% ± 6.8%, 44.1% ± 8.3%, 43.8% ± 6.4% and 47.1% ± 7.1%, to 54.4% ± 4.8%, 57.9% ± 5.8%, 51.4% ± 11.3% and 57.1% ± 4.6% from day 1 to day 7 for S1, S2, S3 and SP respectively. Noteworthy, the increase was less pronounced for S3 as compared to other scaffold configurations for WJ-MSCs.

#### 3.2.1. Distribution and Viability of MSCs on Scaffolds

**Figure 3:**
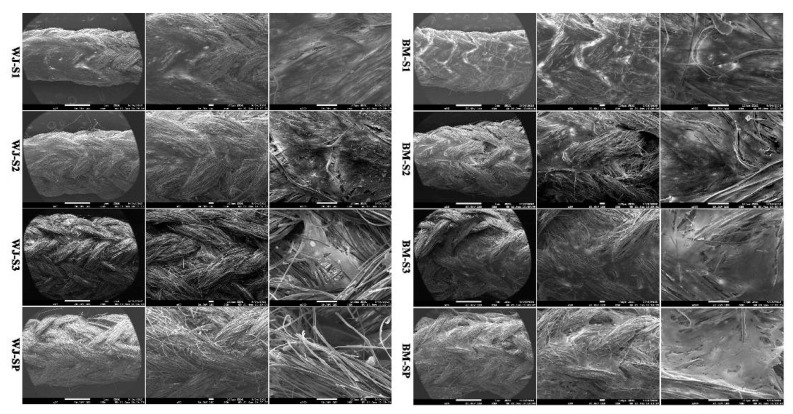
SEM global observations of cellular distribution on S1, S2, S3 and SP scaffolds for WJ-MSCs (left) and BM-MSCs (right).

Analysis of cell behavior on the scaffolds was first performed by SEM. Images showed homogeneous colonization of the scaffolds by WJ-MSCs and BM-MSCs (Figure 3). Both type of MSCs have grown to form cell sheets on all four scaffolds. In accordance with Alamar Blue data, WJ-MSCs were less numerous on S3 compared to the other scaffolds.

**Figure 4:**
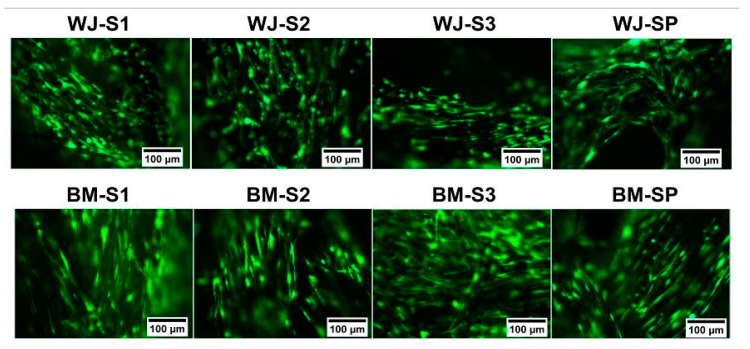
Fluorescent images of live staining of WJ-MSCs (up) and BM-MSCs (down) on the silk and silk/PLCL scaffold (S1: one-layer silk scaffold; S2: two-layer silk scaffold; S3: three-layer silk scaffold; SP: silk/PLCL composite scaffold; green: calcein AM).

Calcein AM was then used to evidence viable cells on scaffolds by fluorescence microscopy. As observed on Figure 4, spindled-shape MSCs have grown along the longitudinal direction of silk fibers. In addition to the silk yarn surface, MSCs were also observed in between fibers of silk yarns. It seemed that BM-MSCs exhibited a more elongated shape with clearer orientation along the fibers than WJ-MSCs.

#### 3.2.2. MSCs Chemotaxis Induced by Scaffolds

To determine if scaffolds exhibited a chemotaxis feature, MSCs were submitted to a transwell migration assay. Using identical conditions, BM-MSCs clearly exhibited higher migration skills than WJ-MSCs over a 24 h period supporting previous results [15]. For both MSCs, chemotaxis was increased for SP scaffolds compared to other scaffold configurations, indicating that the presence of PLCL encouraged the migration of MSCs.

#### 3.2.3. Histology and Immunohistochemistry

**Figure 5:**
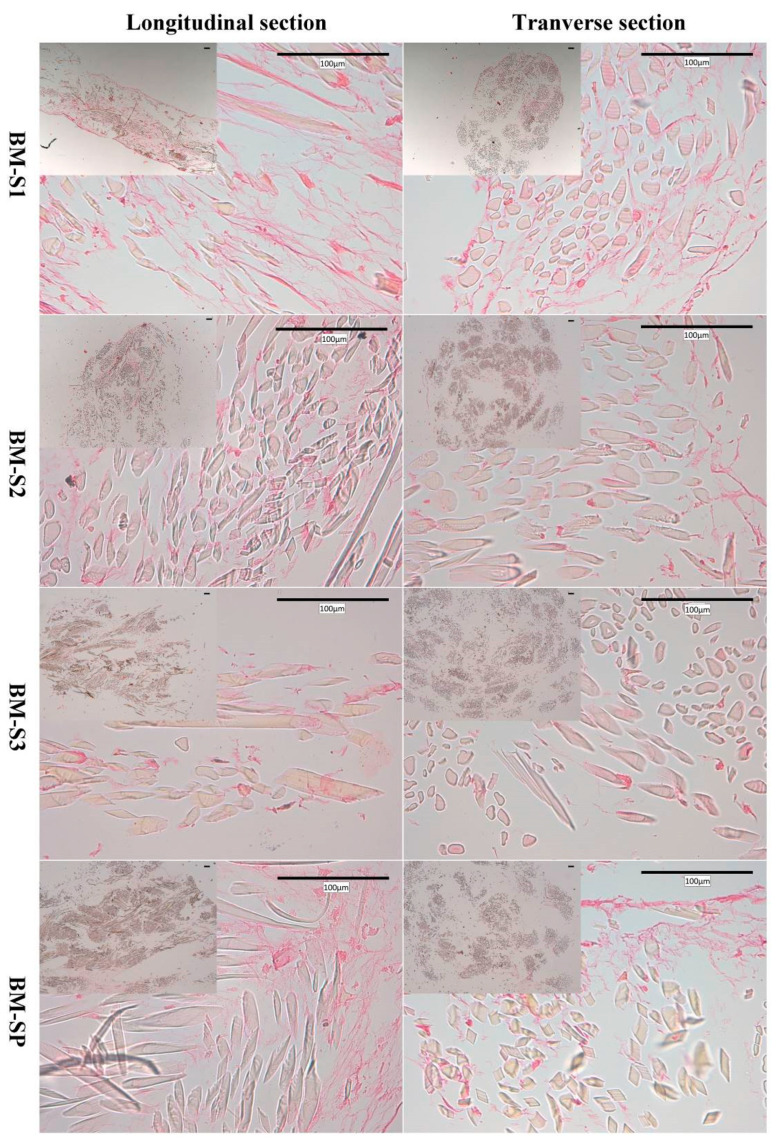
Observation of BM-MSCs on S1, S2, S3 and SP scaffolds from Red Sirius (RS) staining.

Routine histological stainings (hematoxylin/eosin (HES) and Red Sirius (RS)) were performed to provide in situ evidence of MSC presence inside the scaffolds. As an illustration, results are shown in Figure 5 for BM-MSCs (with no observable differences compared to WJ-MSCs). Cell location and extracellular matrix (ECM) synthesis were revealed on the longitudinal and transverse cross sections. According to all the slides of HES staining, cells gathered together and formed cell sheets on the surface of the scaffolds. Moreover, they migrated and proliferated among fibrils in the silk yarns. 

The extracellular matrix (pink) was evidenced both among the yarns and at the periphery of the scaffolds. Transverse sections evidenced that the higher the layer number (from S1 to S3) the lesser the cells were detected. From longitudinal sections, the cell matrix was observed to distribute into the scaffolds and also covered the scaffold surface for S1, S2 and SP, while more ECM was observed in S1, S2 and SP than in the S3 scaffold. Based on the longitudinal slides with RS staining, more collagen was detected in S1 and SP than S2 and S3 for both WJ-MSCs and BM-MSCs, especially compared with S3. On the basis of both HES and RS staining, S1 and SP better supported scaffold colonization and ECM secretion than S2 and S3 scaffolds.

**Figure 6:**
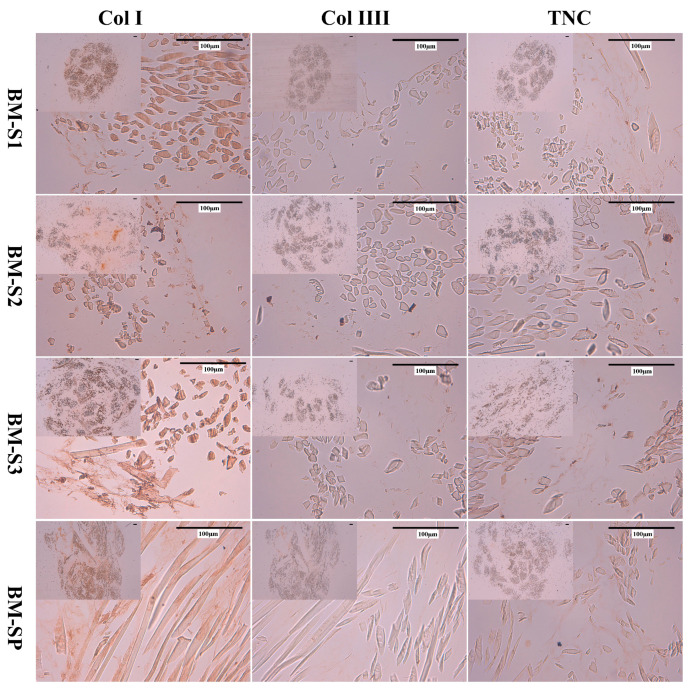
IHC images of BM-MSCs on silk and silk/PLCL scaffolds. (S1: one-layer silk scaffold; S2: two-layer silk scaffold; S3: three-layer silk scaffold; SP: silk/PLCL composite scaffold; Col I: collagen I; Col III: collagen III; TNC: tenascin-c).

Immunohistochemistry (IHC) was used to identify the composition of ECM on MSC-scaffolds, and results are presented in Figure 6. Col I, Col III and TNC were all detected on S1, S2, S3 and SP. More Col I expression was detected than Col III and TNC, since Col I was stained as dark brown network, while Col III and TNC were relatively lighter. According to HES and RS staining, more ECM was detected on S1 and SP than S2 and S3.

## 4. Discussion

We characterized in this work different silk-based scaffolds with multilayered, porous and braided structures and compared for two types of promising cells for tissue engineering. Indeed, silk has been widely used in ligament tissue engineering [8,9,20], especially because of its good mechanical properties and inherent biocompatibility [21,22].

In our previous study [5,15], the PLCL braided scaffold showed promising potential to support the attachment to MSCs. The cell proliferation and migration as well as the extracellular matrix synthesis were promoted, indicating a strong potential for ligament regeneration. However, in the same study we had also observed a premature risk of mechanical failure in the first weeks of implantation due to PLCL [16]. On the contrary, silk was reported to degrade in more than one year, which may not coordinate with the neo-tissue development rate. Based on the suitability of PLCL and silk as biomaterials, the rationale for their association within the novel silk/PLCL composite scaffold reported in the present contribution was to conserve their satisfying biological properties and remedy both the slow degeneration rate of silk as well as the possible mechanical weakness of PLCL, resulting in a biocompatible and biodegradable scaffold for ligament tissue engineering. Composite scaffolds made of silk and PLCL have already been reported for vascular tissue engineering [23], peripheral nerve regeneration [24] and bone regeneration [25] applications. In these studies, silk and PLCL were both fabricated as nanofibers by electrospinning, and cell affinity of PLCL-based scaffolds was improved by the combination with silk.

In the present study, natural silk (after initial degumming) was structurally associated with PLCL without any chemical procedure to avoid the introduction of any chemical agent and save the mechanical properties of natural silk. The previously reported multilayered, braided structure was kept for scaffold fabrication, since this structure was previously reported to mimic native ligament tissue. While different structural combinations could have been imagined, a three-layer silk–PLCL–silk was proposed since the inner core layer plays an essential role on the mechanical response of the braided scaffold [26], and since an intermediate layer of monofilament PLCL fibers should decrease the compactness of the overall structure. A previous non-reported study permitted to conclude that neither the force at failure nor the cellular metabolic activity increased when silk layers exceeded three layers. As a result, the layer number was limited within three layers, and the physical and biological properties of S1, S2, S3 and SP were studied in the present work.

When the number of layers increased, our braided structure showed a higher compacity, except when the PLCL layer was added between two silk layers.

This is due to the monofilament nature of the PLCL layer, in comparison to silk yarns subject to transverse compaction. This may contribute to cellular growth, since porosity has been shown in the literature to be linked to gas or nutrient exchange, local pH stability, tissue ingrowth and cell signaling [27]. In addition to porosity, pore size plays a key role in scaffold design for both cell attachment and cell migration by controlling the surface area available for cell adhesion, and by limiting cell migration if pore size is insufficient [28]. The pore size of scaffolds reported in this study mostly corresponded to the space between silk yarns or between silk yarns and PLCL fibers. In the literature, scaffolds (collagen and polyurethane) with a mean pore size of 20–150 μm have been shown to promote chondrocyte differentiation [29,30], and a larger mean pore size of 250–500 μm has been reported to be favorable for chondrocyte proliferation and ECM synthesis [31,32]. A range of 100–135 μm has been reported [33] to be the optimal pore size for bone growth. Pore size exceeding 300 μm led to osteogenesis; while pore size less than 300 μm encouraged osteochondral ossification [34,35,36]. Pore size in the range 20–120 μm has been reported to be optimal for cell activity towards wound healing and skin tissue engineering [37], while no favorable pore size has been reported for ligament/tendon tissue differentiation. Our numerical computation showed that mean pore size ranged around 30 μm regardless of the scaffold configuration, which is therefore suboptimal compared to the above cited optimal pore size. However, the pore shape in such textile structures is elongated in the direction of the braid, and therefore the proposed image processing algorithms may not be adapted for the proposed structure. The issue of defining an equivalent pore diameter for such elongated pores was not taken into account in the presented procedure based on 2D sections. The assumption of an unsuitable image processing technique is comforted by the fact that, in spite of this insufficient calculated pore size, cell migration was observed on both live/dead images and histological analyses between silk yarns up to the core of the scaffold.

Mechanical properties of the four different scaffold configurations were compared from tensile tests. We observed typical J-shape stress–strain responses for these scaffolds, as described in the literature. The measured values of the apparent tangent moduli were consistent with the literature concerning native ligaments, since it varied from 40 to 100 MPa [38]. The linear tangent moduli were equivalent for S1 and SP, due to the elastic properties at 37 °C largely being lower for PLCL than for silk. The toe tangent moduli tended toward zero as the number of load cycles increased, indicating whether a rearrangement of fibers or viscoelastic effects occurred. This latter proposition is comforted by hysteresis appearing through cycles with a decreased value of the maximum force. Due to the higher forces, the two scaffolds S3 and SP could be considered as better choices in terms of biofunctionality than S1 and S2. Moreover, because of the capacity of PLCL to sustain very large strains, the SP scaffold may decrease the risk of a loss of implant integrity after implantation. 

In order to achieve the final formation of ligamentous tissue, it is crucial to evaluate the proliferation, migration and ECM synthesis of seeded cells [39]. Cell metabolic activity was evaluated in the present study with the Alamar Blue assay for two different types of stromal cells, since no consensus has been formulated concerning the most suitable cell source for ligament tissue engineering. While bone marrow stem cells have been widely used in the past, it has been for instance recently emphasized that the human umbilical cord mesenchymal stem cell may constitute promising alternative candidates [18]. In the present study, we observed that BM-MSC was less sensitive than WJ-MSC to the decrease of scaffold porosity. In fact, BM-MSCs’ metabolic activity increased for all scaffold configurations from day 1 to day 7, while for WJ-MSCs it decreased from S1 to S3 (as the number of layers increased). For the scaffold S3 with the minimal porosity, the WJ-MSCs’ metabolic activity remained almost constant from day 1 to day 7. Anyhow, we observed good adhesion and proliferation for both types of MSCs and cell sheets were observed to cover the surface of scaffolds. From general views obtained by SEM, more WJ-MSCs sheets were observed on S1, while fewer cells were observed on S3, and no significant difference was observed for WJ-MSCs between S2 and SP. For BM-MSCs, more cell sheets were observed on S1, while no significant difference was observed from S2, S3 and SP. According to the live cell staining results, both MSCs migrated and proliferated among the smallest units—fibrils within the yarn. For WJ-MSCs, live cells distributed along the longitudinal direction of silk yarns. As the number of layers increased, scaffolds became more compact, and cells on S2 and S3 seemed to locate at the scaffold surface.

On SP scaffolds, we observed cellular migration and proliferation across both silk and PLCL fibers. We observed a preferential orientation along the fibrous direction for BM-MSCs for each scaffold configuration except D3. Cells were more randomly oriented and distributed for this scaffold with lower porosity.

In order to facilitate tissue regeneration, MSCs should proliferate and migrate within the scaffold to colonize the tissue and create a functional matrix. Cell migration plays therefore a critical role in the success of a tissue engineering strategy. For both types of cells, our results indicate that the migration was encouraged by the insertion of the PLCL layer since the SP scaffold was associated with enhanced cell attraction. The results of HES staining observations revealed that in both longitudinal and transverse sections, cells migrated into the fibrils and that ECM was formed not only on the outer edges of the scaffolds but also inside the scaffolds. We remarked further that for both WJ-MSCs and BM-MSCs, there was more collagen on S1 and SP than on S2 and S3. In particular, collagen was detected in much smaller quantities on S3, which could be explained by the lower porosity of this scaffold that limited cell proliferation and nutrient circulation. The composition of ECM synthesized by WJ-MSCs and BM-MSCs was detected and distinguished with the IHC test. Col I, Col III and TNC were detected in ECM for all the configurations. The ECM synthesis detected with IHC corresponded to the results from HES and RS. More ECM was detected in S1 and SP than S2 and S3, and Col I was more expressed than Col III and TNC. This constitutes an encouraging result since Col I was observed to form more aligned fibers, thus mimicking native tissue.

## 5. Conclusions

From the present data, considering the combination of mechanical, morphological and biological characterizations of the different scaffold configurations, the novel silk/PLCL composite scaffold appeared particularly adapted to ligament tissue engineering compared with the other tested scaffold configurations. The degradation rate of silk/PLCL should be furtherly characterized to complete the properties of the silk/PLCL scaffold since it has been assumed that the presence of PLCL may reduce the degradation time compared to the same silk scaffold. No significant differences between BM-MSCs and WJ-MSCs were observed from the present study, and they both showed satisfying biocompatibility on the composite silk/PLCL scaffold. However, differences were obtained concerning the migration properties as well as the colonization of the S3 scaffold, and additional quantitative characterization related to the ECM synthesis should be performed in the forthcoming studies in order to furtherly compare BM-MSCs and WJ-MSCs. In the future, the silk/PLCL composite scaffold could be modified with LBL technology as described previously [15] for growth factor delivery. The addition of mechanical stimulation using the dedicated bioreactor reported previously [5] to promote cell–scaffold construct differentiation towards ligament tissue also constitute a promising perspective of the present study.
